# The Northern Indiana and Southern Michigan Society

**Published:** 1895-05

**Authors:** 


*Chairman.*

---

### THE NORTHERN INDIANA AND SOUTHERN MICHIGAN SOCIETY.

The eighth semi-annual meeting of the Northern Indiana and Southern Michigan Homœopathic Medical Association was held in Elkhart, Indiana, Thursday, May 2d, 1895, in the Century Club rooms, Dr. W. B. Kreider in the chair. Members present: Drs. G. W. Bowen, Ft. Wayne; I. O. Buchtel, Auburn; E. Franz, Bowen; T. C. Buskirk, White Pigeon; Geo. L. Shoemaker, Nappanee; M. K. and W. B. Kreider, Goshen; W. H. Thomas, A. L. Fisher, A. R. Lieb, and H. A. Mumaw, Elkhart. Visiting physicians: A. D. Smith, Angola; W. F. Lockwood, Wyatt; E. W. Murray, Chicago; S. M. Devor, Elkhart, and Madge D. Mateer, Weihien, China.

The meeting was called to order at 10.45 A. M. by the President, and after roll call, the minutes of the previous meeting were read by the Secretary, Dr. H. A. Mumaw, and approved. Dr. Wm. H. Shaw, Constantine, Mich., was elected to membership.

The necrologist, Dr. Thomas, reported on the death of Dr.

Warren E. Newton, of Ligonier, who was one of the Society's strongest members. He was only thirty-eight years of age. He was a graduate of the Homœopathic Hospital College, of Cleveland, Ohio, and had been a resident of Ligonier for eleven years, where he had a splendid practice, and was surrounded by more friends than most of us enjoy.

The President's annual address was then read. Reports of delegates from other societies were called for. The Treasurer's report showed that the finances of the Association were in a satisfactory condition. Reports of bureaux were next in order. The following papers were read and fully discussed by all the members present: "Eye Injuries," Dr. Kreider; "What are Symptoms, and How do we use them in Prescribing?" by Dr. E. R. McIntyre, Chicago (read by the Secretary); "Dissection and Vivisection," Dr. Green (read by Dr. Fisher); "Psorinum," by Dr. Shaw; "A New Remedy," by Dr. Bowen; "Calipha Indica," by Dr. Fisher; "Gynecological Surgery," by Dr. M. K. Kreider; "Paroxysmal Tachycardia," by Dr. Buchtel; "Remedies Prescribed in Thirteen Hundred Cases," by Dr. Fisher; "Treatment of Catarrh," by Dr. Bowen. A poem entitled "A Contribution to the N. I. and S. M. Homœopathic Medical Association," by Dr. T. P. Wilson, Cleveland, O., was read by the Secretary. A vote of thanks was extended to the doctor for the clever contribution. Reports of cases, by Dr. Buskirk. Dr. Mateer gave her experience in the practice of medicine in China, which was listened to with marked attention.

The Chairman appointed Drs. Mumaw, Fisher, and Thomas a committee on publication to whom the papers read were referred. Chairmen of bureaux for the next meeting were appointed as follows: Surgery, Dr. Turner; ophthalmology, Dr. Beaumont; materia medica, Dr. Franz; practice, Dr. Shaw; gynecology, Dr. M. K. Kreider; pædology, Dr. Lieb.

Election of officers for the ensuing year resulted as follows: President, Dr. Buchtel; first Vice-President, Dr. Thomas; second Vice-President, Dr. Buskirk; Secretary and Treasurer, Dr. Mumaw. Dr. W. A. Whippy was appointed Necrologist.

It was unanimously decided to hold the next meeting at Goshen on the second Tuesday of September next.

Adjourned.

